# Development of a prediction model for suicidal ideation in patients with advanced cancer: A multicenter, real‐world, pan‐cancer study in China

**DOI:** 10.1002/cam4.7439

**Published:** 2024-06-25

**Authors:** Yi He, Ying Pang, Wenlei Yang, Zhongge Su, Yu Wang, Yongkui Lu, Yu Jiang, Yuhe Zhou, Xinkun Han, Lihua Song, Liping Wang, Zimeng Li, Xiaojun Lv, Yan Wang, Juntao Yao, Xiaohong Liu, Xiaoyi Zhou, Shuangzhi He, Yening Zhang, Lili Song, Jinjiang Li, Bingmei Wang, Yang Ke, Zhonghu He, Lili Tang

**Affiliations:** ^1^ Key Laboratory of Carcinogenesis and Translational Research (Ministry of Education/Beijing), Department of Psycho‐oncology Peking University Cancer Hospital and Institute Beijing China; ^2^ Key Laboratory of Carcinogenesis and Translational Research (Ministry of Education/Beijing), Laboratory of Genetics Peking University Cancer Hospital and Institute Beijing China; ^3^ Department of Breast Cancer Radiotherapy, Chinese Academy of Medical Sciences Cancer Hospital Affiliated to Shanxi Medical University Taiyuan China; ^4^ The Fifth Department of Chemotherapy, The Affiliated Cancer Hospital of Guangxi Medical University Guangxi Zhuang Autonomous Region Nanning China; ^5^ Department of Medical Oncology, Cancer Center, West China Hospital Sichuan University Chengdu China; ^6^ Department of Breast Medical Oncology, Shandong Cancer Hospital and Institute Shandong First Medical University and Shandong Academy of Medical Sciences Jinan China; ^7^ Department of Oncology The First Affiliated Hospital of Zhengzhou University Zhengzhou China; ^8^ Department of Oncology Xiamen Humanity Hospital Xiamen China; ^9^ Department of Integrated Chinese and Western Medicine Shaanxi Provincial Cancer Hospital Affiliated to Medical College of Xi'an Jiaotong University Xian China; ^10^ Department of Clinical Spiritual Care, Hunan Cancer Hospital, The Affiliated Cancer Hospital of Xiangya School of Medicine Central South University Changsha China; ^11^ Radiotherapy Center Hubei Cancer Hospital Wuhan China

**Keywords:** advanced cancer, multicenter, pan‐cancer type, risk factor model, suicidal ideation

## Abstract

**Background:**

Patients diagnosed with advanced stage cancer face an elevated risk of suicide. We aimed to develop a suicidal ideation (SI) risk prediction model in patients with advanced cancer for early warning of their SI and facilitate suicide prevention in this population.

**Patients and Methods:**

We consecutively enrolled patients with multiple types of advanced cancers from 10 cancer institutes in China from August 2019 to December 2020. Demographic characteristics, clinicopathological data, and clinical treatment history were extracted from medical records. Symptom burden, psychological status, and SI were assessed using the MD Anderson Symptom Inventory (MDASI), Hospital Anxiety and Depression Scale (HADS), and Patient Health Questionnaire‐9 (PHQ‐9), respectively. A multivariable logistic regression model was employed to establish the model structure.

**Results:**

In total, 2814 participants were included in the final analysis. Nine predictors including age, sex, number of household members, history of previous chemotherapy, history of previous surgery, MDASI score, HADS‐A score, HADS‐D score, and life satisfaction were retained in the final SI prediction model. The model achieved an area under the curve (AUC) of 0.85 (95% confidential interval: 0.82–0.87), with AUCs ranging from 0.75 to 0.95 across 10 hospitals and higher than 0.83 for all cancer types.

**Conclusion:**

This study built an easy‐to‐use, good‐performance predictive model for SI. Implementation of this model could facilitate the incorporation of psychosocial support for suicide prevention into the standard care of patients with advanced cancer.

## 1 INTRODUCTION

Cancer represents a significant global health burden, with both its incidence and mortality rates rising sharply in recent years.^1^, ^2^ Furthermore, research has revealed that cancer patients face nearly double the risk of suicide compared to the general population.^3^ An analysis utilizing data from the surveillance, epidemiology, and end results program found that between 1973 and 2014, 13,311 out of 8,651,569 cancer patients died by suicide, yielding a suicide rate of 28.58 per 100,000 person‐years and a standardized mortality ratio of 4.44 (95% confidence interval, 4.33–4.55).^4^ Notably, patients with metastatic cancer had an even higher suicide rate.^5^

Suicide is a tragic event that is often preventable if appropriate measures are taken to address suicidal ideation (SI) and behavior.^6^ In accordance with the ideation‐to‐action model of suicide, SI is characterized by thoughts, considerations, or plans regarding suicide, thereby sharing a common risk brought by suicidal behavior.^7^, ^8^ Given that suicide behavior can be sudden and urgent, it is imperative to prioritize the identification of SI in the prevention of suicide among cancer patients.

A meta‐analysis revealed that the prevalence of SI among cancer patients in Mainland China has reached nearly 25%.^9^ The causes and risk factors for SI are complex and heterogeneous. Prior research has predominantly concentrated on identifying variables associated with a heightened risk of SI, which is called the traditional “risk‐factor‐identification strategy.” However, an integrated model that incorporates multifaceted predictors to facilitate early screening and identification of patients at risk of SI is lacking.^10^, ^11^ Therefore, it remains challenging for oncology clinicians to identify cancer patients with SI and provide targeted psychological intervention and treatment to prevent suicide.

Therefore, this study conducted a cross‐sectional real‐world study across 10 top‐tier clinical oncology institutions in China, aiming to develop a pragmatic model to screen the risk of SI among advanced cancer patients and to provide a useful tool for early detection and intervention of SI in this population.

## 2 METHODS

### 2.1 Study subjects

Between August 2019 to December 2020, we enrolled consecutively patients aged 18 years and older who had been diagnosed with advanced lung, gastric, breast, liver, colorectal, and esophageal cancer from 10 leading cancer institutions that are situated in 10 representative provinces across China, encompassing the eastern (4 provinces), central (4 provinces), and western regions (2 provinces), with consideration given to both geographical distribution and levels of economic development (Figure S1). Patients with advanced cancer specifically refers to patients who have no treatment options that are curative. Patients with significant communication difficulties, cognitive impairment, or those deemed too frail to complete the questionnaire were excluded.

The study was registered as a clinical trial (ChiCTR1900024957) and has received the approval from the ethics committee of Peking University Cancer Hospital (2019YJZ34). All participants provided informed consent prior to their inclusion in the study.

### 2.2 Clinical data collection

We developed an online real‐time electronic Patient‐Reported Outcome (ePRO) system for patient data collection.^12^ The patient data were uploaded through this platform and monitored in a real‐time manner by designated personnel of this study.

Patients were enrolled upon their initial hospital admission. Following informed consent, all participants completed the Case Report Form including the demographic characteristics (age, sex, smoking history, etc.), clinicopathologic data (diagnosis, cancer stage, etc.), clinical treatment experience (surgery, chemotherapy, radiotherapy, etc.), aided by professionals in each center. The clinical data were gathered from electronic medical records.

### 2.3 Evaluation of psychological status and suicidal ideation

Based on the ePRO system, we assessed the patient's SI, anxiety and depressive symptoms, as well as cancer‐related symptoms by using the MD Anderson Symptom Inventory (MDASI), the Patient Health Questionnaire‐9 (PHQ‐9), the Hospital Anxiety and Depression Scale (HADS) and Insomnia Severity Index (ISI) respectively. Meanwhile, data on self‐reported life satisfaction was also collected.

The PHQ‐9 is a commonly applied screening tool for depressive symptoms and SI, rooted in the Diagnostic and Statistical Manual of Mental Disorders, 4th Edition (DSM‐IV).^13^ The Simplified Chinese version of PHQ‐9 has been well‐validated.^14^ This questionnaire comprises 9 items rated on a 4‐point Likert scale ranging from 0 to 3.

The HADS is a 14 items questionnaire on a 4‐point Likers scale (0–3).^15^ The Chinese version of HADS has been validated and proven reliable.^16^ Anxiety symptoms are assessed in odd‐numbered items, while depressive symptoms are assessed in even‐numbered items. The sum score for each symptom is ranged from 0 to 21, with a higher score indicating more severe anxiety or depressive symptoms.

The MDASI is a questionnaire consisting of 13 items designed for symptom assessment.^17^ The Chinese version has been validated and proven reliable.^18^ Each item scales from 0 (“Nothing”) to 10 (“Most severity”). Symptoms scored 5 to 6 were defined as “moderated,” 7 to 10 as “Severe.” We use MDASI score as a comprehensive factor for symptom burden.

The ISI is a 7 items questionnaire used for measuring the severity of insomnia in the past 2 weeks.^19^ The Chinese version of the ISI has been shown to possess good reliability and validity.^20^ Each item is scored on a scale from 0 (“not at all”) to 4 (“very much”). The total score therefore ranges from 0 to 28, where 0–7 indicates no insomnia, and 8 and higher indicates to insomnia.

### 2.4 Data processing

Variables with more than 10% of missing values were not included in the analysis. The missing values of the rest of the variables were imputed using multiple imputations via chained equations, which generated five complete data sets. For each kind of variable, different imputation methods were applied. Predictive mean matching was used for numerical variables, logistic regression for binary variables, and polytomous logistic regression for categorical variables.^22^ Age was analyzed as a categorical variable, categorized by its percentile, while the number of household members and Eastern Cooperative Oncology Group Performance Status (ECOG PS) were categorized based on accepted cutoff values. To identify the critical candidate predictors, all collected variables were first evaluated using a univariable logistic regression model, and variables that were not clinically relevant to the outcome were excluded. Finally, a total of 23 potential predictive variables were included for subsequent analysis.

### 2.5 Predicted outcome

We defined SI based on item 9 of the PHQ‐9 scale, “Over the last two weeks how often have you been bothered by thoughts that you would be better off dead or of hurting yourself in some way?” The response options for each item of the PHQ‐9 are “not at all” (scoring 0), “several days” (scoring 1), “more than half the days” (scoring 2), or “nearly every day” (scoring 3). Patients who reported such thoughts (i.e., scored 1 or above) were considered as having a SI.

### 2.6 Variable selection and model construction

A two‐step variable selection strategy was used to construct the prediction model. Considering the clinical relevance, we classified all potential predictive variables into four groups, including basic characteristics (age, sex, marital status, occupation, employment status, number of household members, cigarette smoking, psychological counseling experience, personal history of depressive disorder, and family history of depression disorder), disease status (ECOG PS, weight loss, interval between diagnosis, and evaluation), history of treatment (history of previous surgery, chemotherapy, radiotherapy, current treatment, and sides effect of treatment), and self‐reported psychological status (life satisfaction, MDASI score, HADS anxiety [HASD‐A] score, HADS depression [HADS‐D] score, and ISI category). First, the variables in each group were included in a multivariable logistic regression model, and a backward stepwise selection based on Akaike Information Criterion (AIC) was used to identify candidate predictors. Second, the selected candidate predictors from these four groups were once again incorporated into a multivariable logistic model. Taking into account the important risk factors for both SI and cancer type, age and sex were forcibly included as variables in the logistic model. The predictors retained in the final model were determined through AIC‐based backward stepwise elimination and by considering their clinical significance. Each imputed dataset was analyzed and the pooled coefficients, odds ratio (OR), and 95% CIs were obtained by the Rubin's rule.^23^ A nomogram, allowing for visual representation of individual risk of SI, was constructed based on the final model, with points assigned to each predictors determined by their regression coefficients.

### 2.7 Assessment of model performance and validation of the model

We employed the Receiver Operating Characteristic (ROC) curve to assess the final prediction model's ability to discriminate high‐risk individuals for SI. The area under the curve (AUC) was calculated based on the predicted and observed probabilities. Calibration curves were plotted to visually represent the agreement between observed and predicted risk.^24^ To evaluate the accuracy and generalizability of the model, internal validations in each imputed subset of different hospitals and tumor types were performed with R package “psfmi.”^25^

### 2.8 Evaluation of model‐based tailored screening

To assess the application performance of our model, we assumed a hypothetically tailored screening strategy wherein only patients with risk probabilities higher than a specific cutoff value were offered psychological supportive care. The highest predicted probability of achieving the anticipated population coverage in overall patients was selected as the cutoff value. This value was then used to evaluate the application performance of the model in patients with different cancer types. Sensitivity, the detection probability, and the detection probability ratio (as compared to all samples) under each coverage in different datasets were calculated.

### 2.9 Sensitivity analysis

To evaluate the robustness of our main results, we performed a sensitivity analysis where the discriminatory ability of the final model was assessed in the complete dataset without missing values for any predictors. Additionally, we employed the k‐nearest neighbor imputation method to construct and evaluate the model's performance.

All data processing and statistical analysis were performed using Stata 16.0 and R 4.1.2. Statistical significance was set at *p* < 0.05.

## 3 RESULTS

### 3.1 Patient characteristics

A total of 2814 participants with advanced malignant tumors in 10 hospitals qualified for inclusion in the final analysis (Figure S2). Lung cancer was the most prevalent type of cancer diagnosed, comprising 24.0% of all patients, followed by breast, colorectal, stomach, esophageal, and liver cancer. Overall, 598 (21.3%, 95% CI: 19.8%–22.8%) of these patients were identified as having a SI based on item 9 of PHQ‐9. Following a backward stepwise approach, 12 out of 23 variables were selected from the four designated groups, and the distributions of these variables were summarized (Table 1). The median age of the patients in this study was 57, with 58.4% (1643/2814) of them being male. Over half of patients with a household size of 1–3 persons had a history of smoking. Of the included patients, 1132 (40.2%) reported having undergone prior surgery, 1577 (56.0%) had undergone prior chemotherapy, and 440 (15.6%) had undergone radiotherapy. At the time of investigation, 1889 (67.1%) patients were on anti‐cancer therapy (including surgery, chemotherapy, radiotherapy, immunotherapy, and others). The median scores of the MDASI, HADS‐A, HADS‐D and life satisfaction were 19, 5, 5, and 6, respectively.

**TABLE 1:** Selected characteristics of patients with advanced malignant tumor in 10 hospitals, China.

Characteristics	*n* (%) (*N* = 2814)
Cancer site	
Lung	674 (24.0)
Breast	486 (17.3)
Colorectum	444 (15.8)
Stomach	427 (15.2)
Esophagus	405 (14.4)
Liver	354 (12.6)
Multiple	24 (0.9)
Age, years	
<51	751 (26.7)
51–65	1397 (49.6)
≥66	626 (22.2)
Missing	40 (1.4)
Sex	
Male	1643 (58.4)
Female	1147 (40.8)
Missing	24 (0.9)
Number of household member	
1–3	1525 (54.2)
4–6	1009 (35.9)
≥7	106 (3.8)
Missing	174 (6.2)
Cigarette smoking	
No	1611 (57.2)
Yes	1168 (41.5)
Missing	35 (1.2)
History of previous surgery	
No	1546 (54.9)
Yes	1132 (40.2)
Missing	136 (4.8)
History of previous chemotherapy	
No	1047 (37.2)
Yes	1577 (56.0)
Missing	190 (6.8)
History of previous radiotherapy	
No	2171 (77.2)
Yes	440 (15.6)
Missing	203 (7.2)
Current treatment	
No	745 (26.5)
Yes	1889 (67.1)
Missing	180 (6.4)
MDASI score	
Median (IQR)	19 (8, 35)
Missing	101 (3.6)
HADS anxiety score	
Median (IQR)	5 (2, 8)
Missing	31 (1.1)
HADS depression score	
Median (IQR)	5 (2, 9)
Missing	24 (0.9)
Life satisfaction	
Median (IQR)	6 (4, 8)
Missing	6 (0.2)
ISI	
Normal	2502 (88.9)
Insomnia	280 (10.0)
Missing	32 (1.1)

Abbreviations: HADS, hospital anxiety and depression scale; IQR, interquartile range; ISI, insomnia severity index; MDASI, MD Anderson Symptom Inventory.

### 3.2 Prediction model structure

**FIGURE 1:**
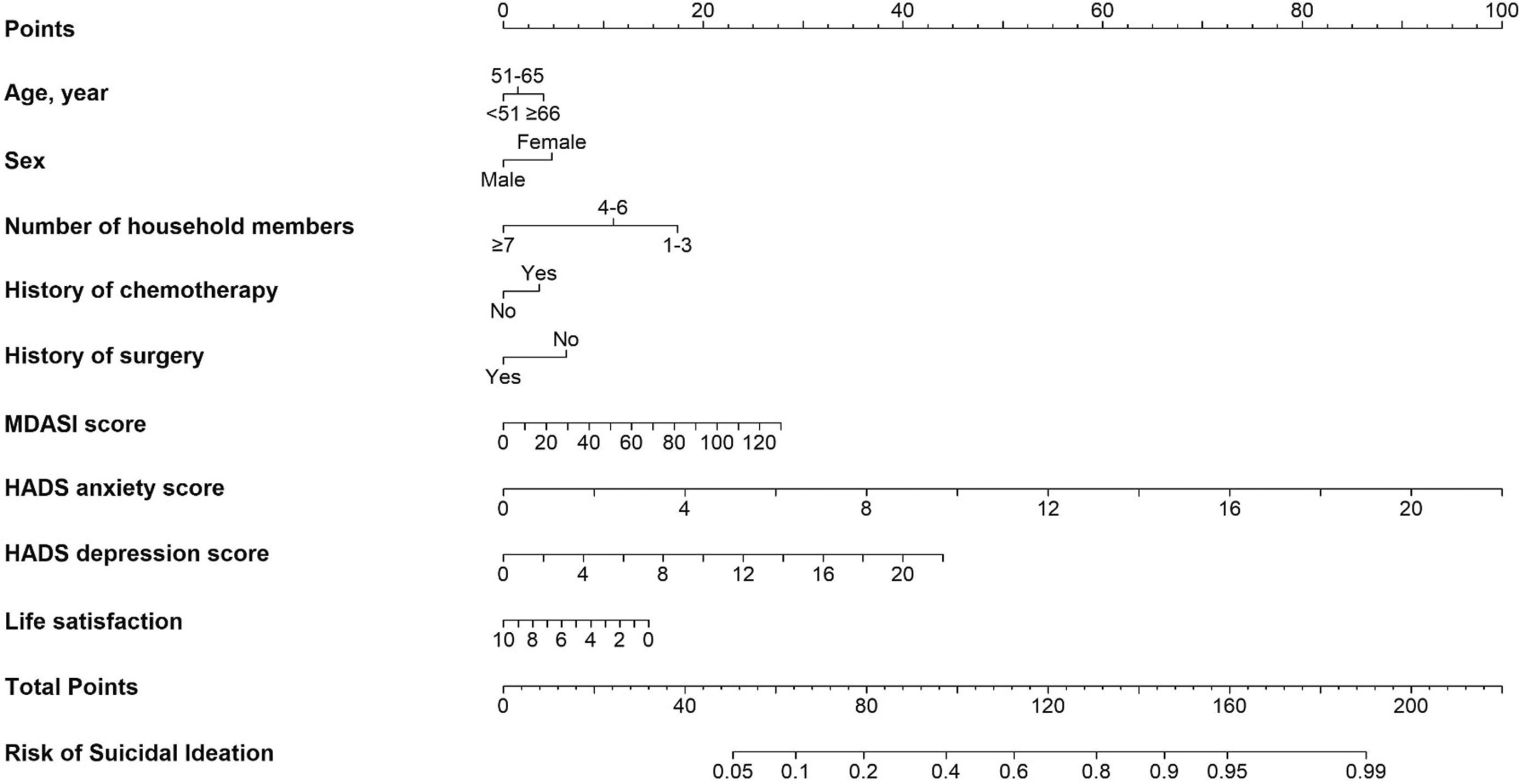
The nomogram for predicting the risk of suicidal ideation in patients with advanced malignant tumors. To calculate the risk of suicidal ideation based on the patient characteristics, first determine the point for each predictor by drawing a vertical line from that predictor to the top points scale. Then sum all of the points and draw a vertical line from the total points scale to the risk line to obtain the risk of suicidal ideation. HADS, hospital anxiety and depression scale; MDASI, MD anderson symptom inventory.

The 12 candidate predictors were fitted into a multivariable logistic model, followed by the AIC‐based backward stepwise selection, resulting in 9 variables retained in the final risk prediction model. The multivariable logistic model showed age ≥66 years old (OR, 1.24 [95% CI, 0.90–1.71]), female (OR,1.30 [95% CI, 1.10–1.58]), had a history of previous chemotherapy (OR,1.21 [95% CI, 0.95–1.55]), higher MDASI score (OR, 1.01[95% CI, 1.01–1.02]), HADS‐A score(OR,1.28 [95% CI, 1.23–1.33]), HADS‐D score(OR, 1.11[95% CI, 1.07–1.16]), associated with a heightened risk of SI, while a greater number of household members (ORs for 4–6 and ≥7 were 0.71[95% CI, 0.55–0.91] and 0.39 [95% CI, 0.19–0.77]), had a history of surgery (OR, 0.71[95% CI, 0.56–0.91]) and more satisfied with life were associated with a heightened risk of SI (OR,0.92 [95% CI, 0.88–0.97]) (Table 2). The nomogram, which incorporates weighting for each factor, was shown in Figure 1. According to points assigned based on the coefficients, the HADS‐A score had the largest effect on the risk of SI.

**TABLE 2:** Structure of the prediction model for predicting suicidal ideation on 2814 patients with advanced malignant tumors from 10 hospitals in China.

Predictors^a^	*n* (%)^b^	Crude OR (95% CI)	Adjusted OR (95% CI)	Adjusted coefficients (95% CI)
Age				
<51	765 (27.2)	Ref.	Ref.	Ref.
51–65	1421 (50.5)	0.98 (0.79, 1.21)	1.08 (0.83, 1.40)	0.08 (−0.19, 0.34)
≥66	628 (22.3)	1.03 (0.80, 1.33)	1.24 (0.90, 1.71)	0.21 (−0.11, 0.54)
Sex				
Male	1658 (58.9)	Ref.	Ref.	Ref.
Female	1156 (41.1)	1.32 (1.10, 1.58)	1.30 (1.03, 1.63)	0.26 (0.03, 0.49)
Number of household members				
1–3	1639 (58.2)	Ref.	Ref.	Ref.
4–6	1068 (38.0)	0.85 (0.70, 1.04)	0.71 (0.55, 0.91)	−0.35 (−0.60, −0.10)
≥7	107 (3.8)	0.57 (0.32, 1.01)	0.39 (0.19, 0.77)	−0.95 (−1.64, −0.26)
History of previous chemotherapy				
No	1104 (39.2)	Ref.	Ref.	Ref.
Yes	1710 (60.8)	1.03 (0.84, 1.24)	1.21 (0.95, 1.55)	0.19 (−0.05, 0.44)
History of previous surgery				
No	1638 (58.2)	Ref.	Ref.	Ref.
Yes	1176 (41.8)	0.59 (0.49, 0.72)	0.71 (0.56, 0.91)	−0.34 (−0.58, −0.1)
MDASI score				
Continuous (median, IQR)	19 (9, 35)	1.04 (1.03, 1.04)	1.01 (1.01, 1.02)	0.01 (0.01, 0.02)
HADS anxiety score				
Continuous	5 (2, 8)	1.42 (1.38, 1.47)	1.28 (1.23, 1.33)	0.25 (0.21, 0.29)
HADS depression score				
Continuous	5 (2, 9)	1.32 (1.28, 1.36)	1.11 (1.07, 1.16)	0.11 (0.07, 0.14)
Life satisfaction				
Continuous	6 (4, 8)	0.80 (0.78, 0.83)	0.92 (0.88, 0.97)	−0.08 (−0.13, −0.03)

Abbreviations: HADS, Hospital Anxiety and Depression Scale; IQR, interquartile range; MDASI, MD Anderson Symptom Inventory.

^a^ A two‐phase selection based on logistic regression model and backward elimination under Akaike Information Criterion (AIC) was used to determine the final predictor panel. Only variables included in the final prediction model are shown in this table.

^b^ The 3rd imputed dataset was used to calculate the proportion and median value of predictors.

### 3.3 Performance of the prediction model

**FIGURE 2:**
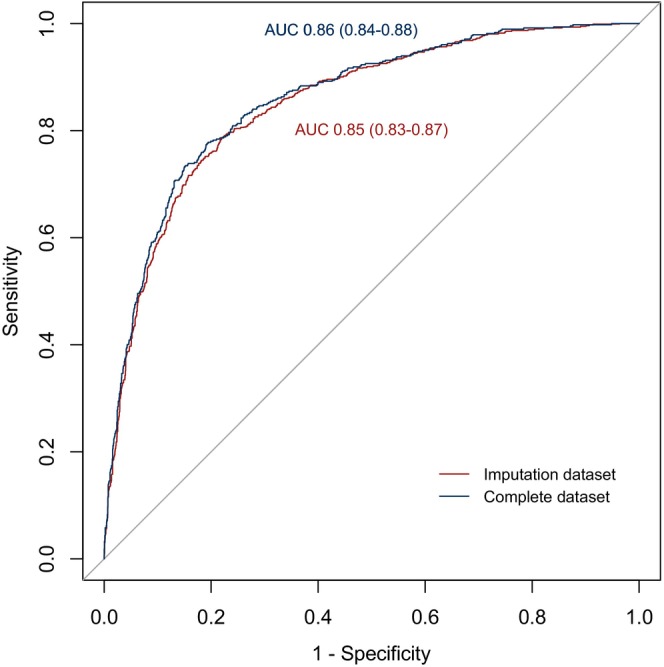
Receiver operating characteristic (ROC) curves of the risk prediction model for suicidal ideation in multiple imputed dataset (red color) and complete dataset (blue color) among patients with advanced malignant tumor.

**FIGURE 3:**
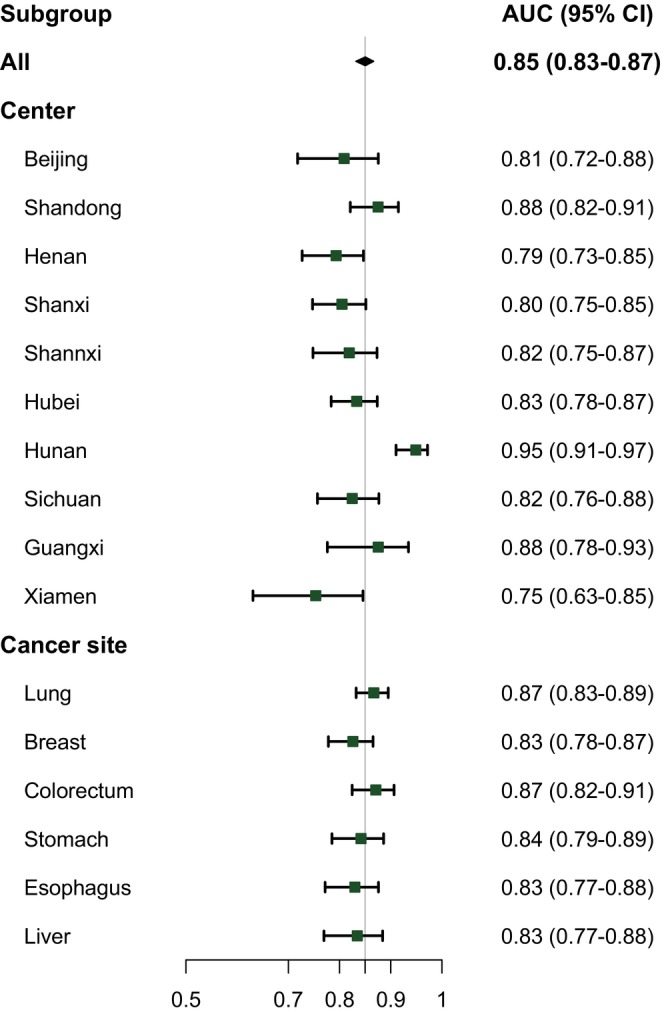
The area under curves (AUCs) of the risk prediction model in the internal validation set within each hospital and cancer site.

The AUC of this model was 0.85 (95% CI: 0.82–0.87) (Figure 2). When applied to different subsets according to study centers and cancer types, the predictive model continued to demonstrate ideal performance, with AUCs ranging from 0.75 to 0.95 across 10 hospitals and higher than 0.83 for all cancer types (Figure 3). Calibration plots showed optimal agreement between model‐predicted and actual probability for SI in overall patients and patients with different cancers (Figure S3).

### 3.4 Application of the prediction model

**FIGURE 4:**
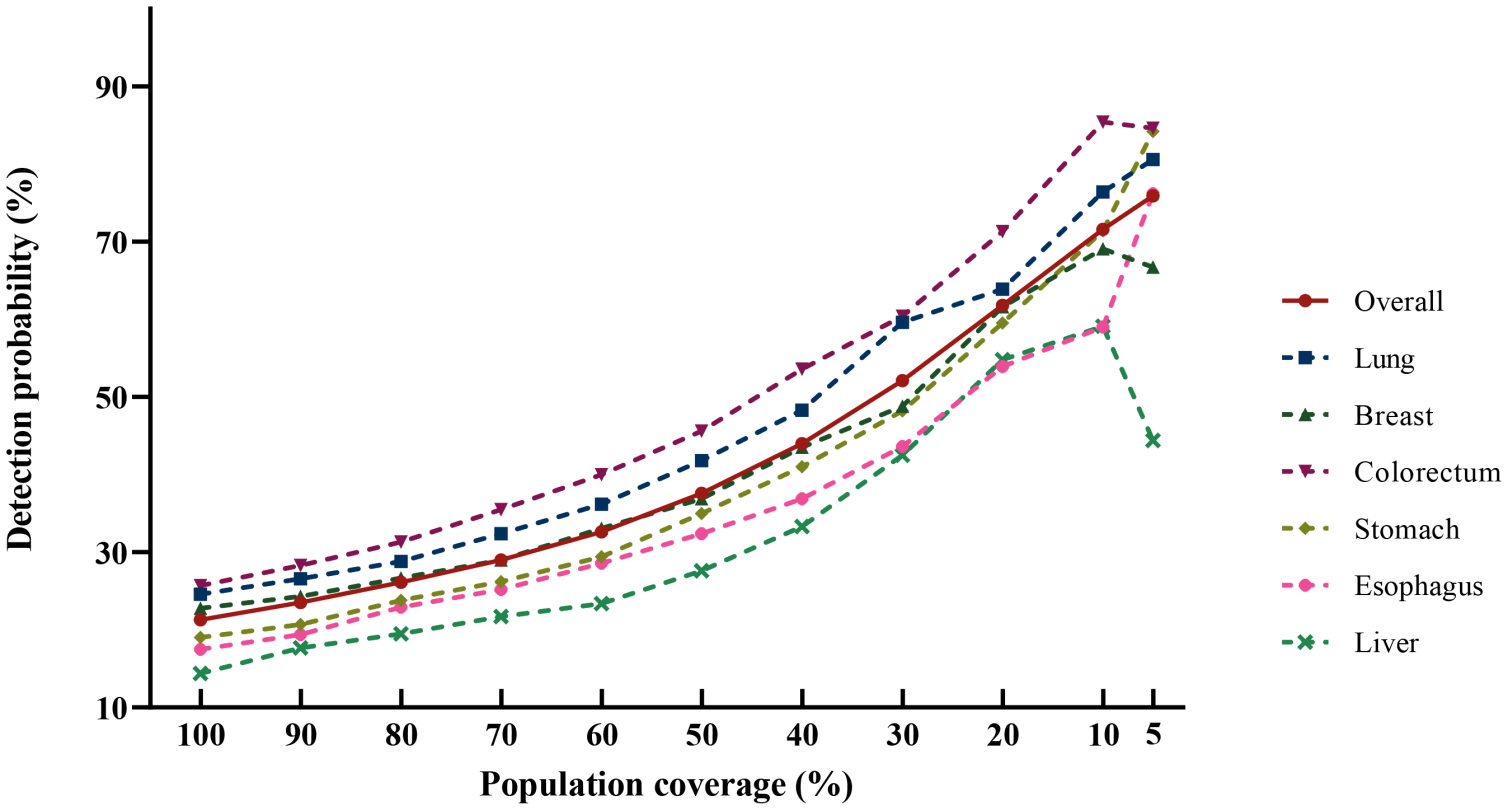
Detection rate of assumptive tailored screening with different cutoffs when the established prediction model was applied in all patients and patients with different cancer site.

The application performance of our model under different workloads of psychological supportive care was accessed by the assumed hypothetic tailored screening strategy wherein “high‐risk” patients were selected for the referral. There was a similar trend observed across all patients and six types of cancer, where the detection probability increased greatly as the cutoff value was raised (Figure 4; Table S2), which demonstrated the capability of the model to identify and enrich high‐risk patients with SI. For instance, in the scenario where only patients at the top 10% risk level would be referred for psychological supportive care, the detection probability would be over three‐fold higher than the universal screening in any dataset. When we expected to cover more cases, like at least 80% of all cases (i.e., the sensitivity above 80%), only the top 40% of patients in the overall dataset were needed to refer (2.1‐fold increase in detection probability), and similar results were also found in patients with different cancers.

### 3.5 Sensitivity analysis

Sensitivity analysis was conducted in the complete dataset, in which 2303 (81.8%) available patients were included. The discrimination of the prediction model was consistent with the main analysis (AUC = 0.86, 95% CI: 0.84–0.88) (Figure 2). The model's structure and the corresponding AUC in the k‐nearest neighbor imputated datasets demonstrated comparable results (Table S3).

## 4 DISCUSSION

Suicide is a significant problem among cancer patients, and preventing it is a challenging task in clinical oncology. In accordance with the ideation‐to‐action framework of suicide, early identification and addressing SI is crucial to prevent the fatal outcome of suicide. Based on this, we examined nearly 3000 patients with advanced stages of six common cancers from 10 cancer centers in China, and for the first time, we built an easy‐to‐use model predicting the risk of SI among individuals diagnosed with advanced cancer. This model was established using cross‐sectional data and is not intended as a prognostic model, but rather as a diagnostic tool to identify high‐risk individuals and facilitate timely psychological intervention. By applying this model to clinical practice, we hope to provide a warning of SI and mitigate the risk of suicide among cancer patients.

Our model indicated that older and female patients had a higher risk of SI compare to younger and male patients. These results contradict with the findings of a US research^19^ that greater suicide risk is correlated with patients in male, age group 60–69. One reason for the discrepancy in results may be due to the heterogeneous of subjects in two studies. In Chinese society, the older and female patients may be more vulnerable.^20^ With advanced cancer, this population may receive less social support and need more attention. In addition, data from current study indicate that the risk of SI was lower in patients with fewer household members. This result is consistent with the research conducted by Zhou et al. which suggests that family adaptability and cohesion serve as protective factors against SI in Chinese cancer patients.^21^ These findings emphasized that adequate social and family support is very important for patients in the face of advanced cancer. Facilitating social and family support should be one of the interventions to avoid developing SI in this population.

The history of anti‐cancer treatments, such as chemotherapy and surgery, was retained in the final model, in which, the history of chemotherapy was associated with an elevated risk of SI. This agreed with the findings of previous studies that the side effects of chemotherapy had huge impact on patients in both physical and psychological ways.^26^, ^27^ However, the current study indicated that the history of cancer surgery was the protective factor of SI. It might be due to the better expectation of disease prognosis among patients who underwent radical surgery. This finding provides new perspective on the correlation between SI and cancer treatment. Areas for future research include the psychological impact of surgery in postoperative period among cancer patients.

Consistent with previous studies, physical symptoms and psychological distress predicted an increased likelihood of SI among patients with advanced cancer.^28^, ^29^ Previous research suggested that quality of life could act as a mediator in the association between symptom burden and SI,^4^ and thus active symptom control, for example, analgesia, to improve the overall life quality should be essential to reduce SI. From the nomogram, anxiety and depression contributed the most to the model, which underlined the important role of anxiety/depression in the formation of SI. Therefore, it is imperative to enhance the screening, evaluation, and management of cancer‐related symptoms and psychological distress in oncology clinical practice. In most cancer patients, symptoms such as pain, distress, or fatigue usually present as a cluster,^30^ and one single symptom cannot represent the overall symptom burden. In this study, we used the total score of MDASI as a variable to predict SI, which mainly represents the overall symptom burden of patients.

In the present study, our model showed favorable accuracy (AUC = 0.83) in the overall dataset and desired robustness and homogeneity were found in the 16 sub‐cohorts including the 10 study centers and six cancer types. Calibration plots for the risk of SI overall and each cancer dataset were all close to the 45‐degree line. Moreover, this model yielded consistent risk enrichment trends in patients with different regions and cancer types, which suggested the ideal adaptability and generalizability of the model when applied to different cancer patients and regions.

The model established in this study could serve as a valuable resource for oncology clinicians to identify advanced cancer patients at high‐risk of SI, facilitating more precise referrals. For example, if the cutoff is set at 0.709, there will be 17.9% of advanced cancer patients have been diagnosed as the high‐risk subgroup for SI, which is a 3.6‐fold increase over the average for the entire sample. When it was applied in real‐word scenarios, different cut‐off values should be adopted according to their psychosocial service capability.

In current clinical practice, suicide prevention is mostly reactive, the intervention is provided when the suicide behavior occurs. However, implementing this predictive model to identify patients at a heightened risk of SI and to give them intervention timely could change the reactive model into a proactive one, which could also facilitate the integration of psychosocial care into standard cancer care practices, to identify the patients' psychosocial distress more actively rather than waiting for their report passively, and to let more patients access the psychosocial care they needed.

We acknowledge that there may be information bias introduced into the data collection, as SI was assessed using the suicidal item within the PHQ‐9 scale rather than the Columbia‐Suicide Severity Rating Scale Screener (C‐SSRS), which is widely regarded as the gold standard for evaluating SI^31^ However, in traditional Chinese culture, the investigation of SI may cause discomfort or even resistance among participants due to the sensitivity and taboo of death. Therefore, hiding the suicide information among other items of the comprehensive scale may reduce the psychological burden and defense mechanism of the respondents and enable more real information to be collected. Moreover, the item‐9 of PHQ‐9 is frequently utilized in screening of SI among cancer pateints.^32^, ^33^ Although the C‐SSRS is the standard for assessing suicide risk, it also has some limitations, such as lower sensitivity to suicide risk.^34^

There are two limitations in this study. First, although 10 cancer centers were included in this multicenter study, the sample size of each center is still relatively limited, and further validation of our model is still needed in a larger sample size. Second, there is a lack of full life cycle follow‐up, so the ability of this model to predict actual suicidal behavior is still uncertain. Third, some potential variables, such as urban/rural residence status and financial burden, were not collected in this study. The multi‐center sites involved in this study are all provincial‐level cancer centers. Patients seeking treatment at these centers generally have economic means beyond the basic threshold, and currently, medical insurance covers both urban and rural areas. Financial burden is a sensitive variable that is not easily accessible. These variables should be considered in future studies.

In summary, we built an easy‐to‐use, good‐performance prediction model for SI among patients with common advanced cancers based on multi‐center real‐world data and also proposed the criteria for SI risk levels. A user‐friendly online prediction tool (https://riskofsi.shinyapps.io/rpublish/) has been developed, featuring an interactive interface for parameter input and graphic results display (Figure S4). This work provides a useful tool for future clinical oncology multidisciplinary care, to improve cancer patients' quality of life and reduce the risk of suicide behavior.

## AUTHOR CONTRIBUTIONS

**Yi He:** Conceptualization (equal); data curation (lead); formal analysis (equal); investigation (equal); project administration (equal); writing – original draft (equal); writing – review and editing (equal). **Ying Pang:** Data curation (equal); formal analysis (equal); investigation (equal); project administration (equal); writing – original draft (equal); writing – review and editing (equal). **Wenlei Yang:** Formal analysis (equal); methodology (equal); validation (equal); writing – original draft (equal); writing – review and editing (equal). **Zhongge Su:** Writing – original draft (equal); writing – review and editing (equal). **Yu Wang:** Data curation (equal); writing – review and editing (equal). **Yongkui Lu:** Data curation (equal); writing – review and editing (equal). **Yu Jiang:** Data curation (equal); writing – review and editing (equal). **Yuhe Zhou:** Data curation (equal); investigation (equal); software (equal). **Xinkun Han:** Data curation (equal); investigation (equal); software (equal). **Lihua Song:** Data curation (equal); project administration (equal). **Liping Wang:** Data curation (equal); project administration (equal). **Zimeng Li:** Investigation (equal); project administration (equal). **Xiaojun Lv:** Data curation (equal). **Yan Wang:** Investigation (equal); project administration (equal). **Juntao Yao:** Data curation (equal). **Xiaohong Liu:** Data curation (equal). **Xiaoyi Zhou:** Data curation (equal). **Shuangzhi He:** Investigation (equal); project administration (equal). **Yening Zhang:** Project administration (equal). **Lili Song:** Project administration (equal). **Jinjiang Li:** Project administration (equal). **Bingmei Wang:** Project administration (equal). **Yang Ke:** Supervision (supporting); writing – review and editing (equal). **Zhonghu He:** Data curation (equal); formal analysis (equal); methodology (equal); validation (equal); visualization (equal); writing – original draft (equal); writing – review and editing (equal). **Lili Tang:** Conceptualization (lead); funding acquisition (equal); resources (lead); supervision (lead); writing – original draft (equal); writing – review and editing (equal).

## FUNDING INFORMATION

This study was supported by China Association of Gerontology and Geriatrics.

## CONFLICT OF INTEREST STATEMENT

All authors have disclosed that they have no conflicts of interest.

## Supporting information


Data S1.

## Data Availability

The data supporting the findings of this study are available from the corresponding author upon reasonable request.
