# Supplementary material for: Development of a prediction model for suicidal ideation in patients with advanced cancer: A multicenter, real‐world, pan‐cancer study in China
Source: Cancer Med. 2024 Jun 25;13(12):e7439. doi: 10.1002/cam4.7439 (PMC11196995; doi:10.1002/cam4.7439)

| Table S1. Characteristics of patients with advanced malignant tumors in ten hospitals, China. | |
| --- | --- |
| **Characteristics** | **n (%) (N = 2814)** |
| **Cancer site** |  |
| Lung | 674 (24.0) |
| Breast | 486 (17.3) |
| Colorectum | 444 (15.8) |
| Stomach | 427 (15.2) |
| Esophagus | 405 (14.4) |
| Liver | 354 (12.6) |
| Multiple | 24 (0.9) |
| **Age, years** |  |
| <51 | 751 (26.7) |
| 51-65 | 1397 (49.6) |
| ≥66 | 626 (22.2) |
| Missing | 40 (1.4) |
| **Sex** |  |
| Male | 1643 (58.4) |
| Female | 1147 (40.8) |
| Missing | 24 (0.9) |
| **Marital status** |  |
| Married | 2677 (95.1) |
| Other | 108 (3.8) |
| Missing | 29 (1.0) |
| **Occupation** |  |
| Farmer | 1269 (45.1) |
| Enterprise personnel | 713 (25.3) |
| Other | 782 (27.8) |
| Missing | 50 (1.8) |
| **Employment status** |  |
| Full-time | 1542 (54.8) |
| Part-time | 1243 (44.2) |
| Missing | 29 (1.0) |
| **Number of household members** |  |
| 1-3 | 1525 (54.2) |
| 4-6 | 1009 (35.9) |
| ≥7 | 106 (3.8) |
| Missing | 174 (6.2) |
| **Received any psychological counseling service** | |
| No | 2729 (97.0) |
| Yes | 23 (0.8) |
| Missing | 62 (2.2) |

| **Table S1 (continued)** | |
| --- | --- |
| **Characteristics** | **n (%) (N = 2814)** |
| **Personal history of depression disorder** | |
| No | 2747 (97.6) |
| Yes | 7 (0.2) |
| Missing | 60 (2.1) |
| **Family history of depression disorder** | |
| No | 2739 (97.3) |
| Yes | 24 (0.9) |
| Missing | 51 (1.8) |
| **Cigarette smoking** |  |
| No | 1611 (57.2) |
| Yes | 1168 (41.5) |
| Missing | 35 (1.2) |
| **ECOG score** |  |
| 0-1 | 2474 (87.9) |
| 2-4 | 340 (12.1) |
| **Weight loss** |  |
| <5% | 1914 (68.0) |
| 5%-<10% | 542 (19.3) |
| ≥10% | 213 (7.6) |
| Missing | 145 (5.2) |
| **Interval between diagnosis and evaluation, years** | |
| 0 | 1779 (63.2) |
| 1-2 | 451 (16.0) |
| ≥3 | 363 (12.9) |
| Missing | 221 (7.9) |
| **History of previous surgery** |  |
| No | 1546 (54.9) |
| Yes | 1132 (40.2) |
| Missing | 136 (4.8) |
| **History of previous chemotherapy** |  |
| No | 1047 (37.2) |
| Yes | 1577 (56.0) |
| Missing | 190 (6.8) |
| **History of previous radiotherapy** |  |
| No | 2171 (77.2) |
| Yes | 440 (15.6) |
| Missing | 203 (7.2) |

| **Table S1 (continued)** | |
| --- | --- |
| **Characteristics** | **n (%) (N = 2814)** |
| **Current treatment** |  |
| No | 745 (26.5) |
| Yes | 1889 (67.1) |
| Missing | 180 (6.4) |
| **Side effects of treatment** |  |
| Median (IQR) | 3.00 (1.00, 4.00) |
| Missing | 38 (1.4) |
| **MDASI score^*^** |  |
| Median (IQR) | 19 (8, 35) |
| Missing | 101 (3.6) |
| **Life satisfaction** |  |
| Median (IQR) | 6 (4, 8) |
| Missing | 6 (0.2) |
| **HADS anxiety score** |  |
| Median (IQR) | 5 (2, 8) |
| Missing | 31 (1.1) |
| **HADS depression score** |  |
| Median (IQR) | 5 (2, 9) |
| Missing | 24 (0.9) |
| **ISI^†^** |  |
| Normal | 2502 (88.9) |
| Insomnia | 280 (10.0) |
| Missing | 32 (1.1) |
| *MDASI score was calculated by summing up the points from 13 core symptom severity items (pain, fatigue, nausea, sleep disturbance, distress, shortness of breath, difficulty remembering, poor appetite, drowsiness, dry mouth, sadness, vomiting, and numbness).  †Clinically significant insomnia was defined as an ISI total score ≥ 15.  Abbreviations: HADS, Hospital Anxiety and Depression Scale; IQR, interquartile range; ISI, Insomnia Severity Index; MDASI, M.D. Anderson Symptom Inventory. | |

| Table S2. Evaluation of application performance of the prediction model for different screening coverages to detect suicidal ideation. | | | | | | | | | | | | | | | |
| --- | --- | --- | --- | --- | --- | --- | --- | --- | --- | --- | --- | --- | --- | --- | --- |
| **Cutoffs** | **Overall** | | |  | **Lung** | | |  | **Breast** | | |  | **Colorectum** | | |
|  | **Population coverage (sensitivity) (%)** | **Detection probability (%)** | **Detection probability ratio (compared to all samples)** |  | **Population coverage (sensitivity) (%)** | **Detection probability (%)** | **Detection probability ratio (compared to all samples)** |  | **Population coverage (sensitivity) (%)** | **Detection probability (%)** | **Detection probability ratio (compared to all samples)** |  | **Population coverage (sensitivity) (%)** | **Detection probability (%)** | **Detection probability ratio (compared to all samples)** |
| 0.7094087 | 5.0 (17.9) | 75.9 | 3.6 |  | 5.3 (17.5) | 80.6 | 3.3 |  | 5.6 (16.2) | 66.7 | 2.9 |  | 5.9 (19.3) | 84.6 | 3.3 |
| 0.5775469 | 10.0 (33.8) | 71.6 | 3.4 |  | 10.7 (33.1) | 76.4 | 3.1 |  | 11.3 (34.2) | 69.1 | 3.0 |  | 10.8 (36.0) | 85.4 | 3.3 |
| 0.3916926 | 20.0 (58.2) | 61.8 | 2.9 |  | 23.4 (60.8) | 63.9 | 2.6 |  | 20.4 (55.0) | 61.6 | 2.7 |  | 22.7 (63.2) | 71.3 | 2.8 |
| 0.2581737 | 30.0 (73.6) | 52.1 | 2.4 |  | 32.3 (78.3) | 59.6 | 2.4 |  | 33.3 (71.2) | 48.8 | 2.1 |  | 33.6 (78.9) | 60.4 | 2.4 |
| 0.1765787 | 40.0 (82.9) | 44.0 | 2.1 |  | 44.2 (86.7) | 48.3 | 2.0 |  | 43.0 (82.0) | 43.5 | 1.9 |  | 41.2 (86.0) | 53.6 | 2.1 |
| 0.1167362 | 50.0 (88.5) | 37.6 | 1.8 |  | 53.6 (91.0) | 41.8 | 1.7 |  | 55.1 (89.2) | 36.9 | 1.6 |  | 50.9 (90.4) | 45.6 | 1.8 |
| 0.0750961 | 60.0 (92.1) | 32.6 | 1.5 |  | 63.5 (93.4) | 36.2 | 1.5 |  | 65.2 (94.6) | 33.1 | 1.5 |  | 59.7 (93.0) | 40.0 | 1.6 |
| 0.0501863 | 70.0 (95.7) | 29.0 | 1.4 |  | 74.2 (97.6) | 32.4 | 1.3 |  | 75.1 (95.5) | 29.0 | 1.3 |  | 68.5 (94.7) | 35.5 | 1.4 |
| 0.0326737 | 80.0 (98.3) | 26.1 | 1.2 |  | 85.0 (99.4) | 28.8 | 1.2 |  | 84.2 (98.2) | 26.7 | 1.2 |  | 81.3 (99.1) | 31.3 | 1.2 |
| 0.0203445 | 90.0 (99.3) | 23.5 | 1.1 |  | 92.7 (100.0) | 26.6 | 1.1 |  | 93.8 (100.0) | 24.3 | 1.1 |  | 89.9 (99.1) | 28.3 | 1.1 |
| 0.004654 | 100.0 (100.0) | 21.3 | 1.0 (Ref.) |  | 100.0 (100.0) | 24.6 | 1.0 (Ref.) |  | 100.0 (100.0) | 22.8 | 1.0 (Ref.) |  | 100.0 (100.0) | 25.7 | 1.0 (Ref.) |

| **Table S2 (continued)** | | | | | | | | | | | | | | | |
| --- | --- | --- | --- | --- | --- | --- | --- | --- | --- | --- | --- | --- | --- | --- | --- |
| **Cutoffs** | **Overall** | | |  | **Stomach** | | |  | **Esophagus** | | |  | **Liver** | | |
|  | **Population coverage (sensitivity) (%)** | **Detection probability (%)** | **Detection probability ratio (compared to all samples)** |  | **Population coverage (sensitivity) (%)** | **Detection probability (%)** | **Detection probability ratio (compared to all samples)** |  | **Population coverage (sensitivity) (%)** | **Detection probability (%)** | **Detection probability ratio (compared to all samples)** |  | **Population coverage (sensitivity) (%)** | **Detection probability (%)** | **Detection probability ratio (compared to all samples)** |
| 0.7094087 | 5.0 (17.9) | 75.9 | 3.6 |  | 4.4 (19.8) | 84.2 | 4.4 |  | 5.2 (22.5) | 76.2 | 4.4 |  | 2.5 (7.8) | 44.4 | 3.1 |
| 0.5775469 | 10.0 (33.8) | 71.6 | 3.4 |  | 9.8 (37.0) | 71.4 | 3.8 |  | 9.6 (32.4) | 59.0 | 3.4 |  | 6.2 (25.5) | 59.1 | 4.1 |
| 0.3916926 | 20.0 (58.2) | 61.8 | 2.9 |  | 18.5 (58.0) | 59.5 | 3.1 |  | 18.8 (57.7) | 53.9 | 3.1 |  | 11.9 (45.1) | 54.8 | 3.8 |
| 0.2581737 | 30.0 (73.6) | 52.1 | 2.4 |  | 26.7 (67.9) | 48.2 | 2.5 |  | 28.9 (71.8) | 43.6 | 2.5 |  | 20.6 (60.8) | 42.5 | 3.0 |
| 0.1765787 | 40.0 (82.9) | 44.0 | 2.1 |  | 36.5 (79.0) | 41.0 | 2.2 |  | 38.8 (81.7) | 36.9 | 2.1 |  | 31.4 (72.5) | 33.3 | 2.3 |
| 0.1167362 | 50.0 (88.5) | 37.6 | 1.8 |  | 46.1 (85.2) | 35.0 | 1.8 |  | 46.4 (85.9) | 32.4 | 1.9 |  | 42.9 (82.4) | 27.6 | 1.9 |
| 0.0750961 | 60.0 (92.1) | 32.6 | 1.5 |  | 58.1 (90.1) | 29.4 | 1.5 |  | 55.3 (90.1) | 28.6 | 1.6 |  | 53.1 (86.3) | 23.4 | 1.6 |
| 0.0501863 | 70.0 (95.7) | 29.0 | 1.4 |  | 68.9 (95.1) | 26.2 | 1.4 |  | 66.7 (95.8) | 25.2 | 1.4 |  | 61.3 (92.2) | 21.7 | 1.5 |
| 0.0326737 | 80.0 (98.3) | 26.1 | 1.2 |  | 76.8 (96.3) | 23.8 | 1.3 |  | 75.6 (98.6) | 22.9 | 1.3 |  | 70.9 (96.1) | 19.5 | 1.4 |
| 0.0203445 | 90.0 (99.3) | 23.5 | 1.1 |  | 89.2 (97.5) | 20.7 | 1.1 |  | 88.9 (98.6) | 19.4 | 1.1 |  | 81.4 (100.0) | 17.7 | 1.2 |
| 0.004654 | 100.0 (100.0) | 21.3 | 1.0 (Ref.) |  | 100.0 (100.0) | 19.0 | 1.0 (Ref.) |  | 100.0 (100.0) | 17.5 | 1.0 (Ref.) |  | 100.0 (100.0) | 14.4 | 1.0 (Ref.) |

| **Table S3. The predictors and area under curves using k-nearest neighbor imputation dataset.** | | | |
| --- | --- | --- | --- |
| **Imputation method** | **k*** | **Predictors** | **AUC (95% CI)** |
| kNN† | 10 | Age, sex, number of household members, history of previous surgery, MDASI score, HADS anxiety score, HADS depression score, life satisfaction | 0.8537 (0.8367-0.8706 |
| kNN | 20 | Age, sex, number of household members, history of previous chemotherapy, history of previous surgery, MDASI score, HADS anxiety score, HADS depression score, life satisfaction | 0.8542 (0.8373-0.8712) |
| kNN | 30 | Age, sex, number of household members, history of previous chemotherapy, history of previous surgery, MDASI score, HADS anxiety score, HADS depression score, life satisfaction | 0.8545 (0.8375-0.8714) |
| Abbreviations: kNN, k-nearest nearest neighbor.  *k indicated the numbers of nearest neighbors used.  †Compared to the final model, predictors selected using the k=10 method without the history of previous chemotherapy. | | | |

**Figure S1. Geographic distribution of the participating hospitals comprising of provincial cancer hospitals (red color) and tertiary general hospitals (blue color).**


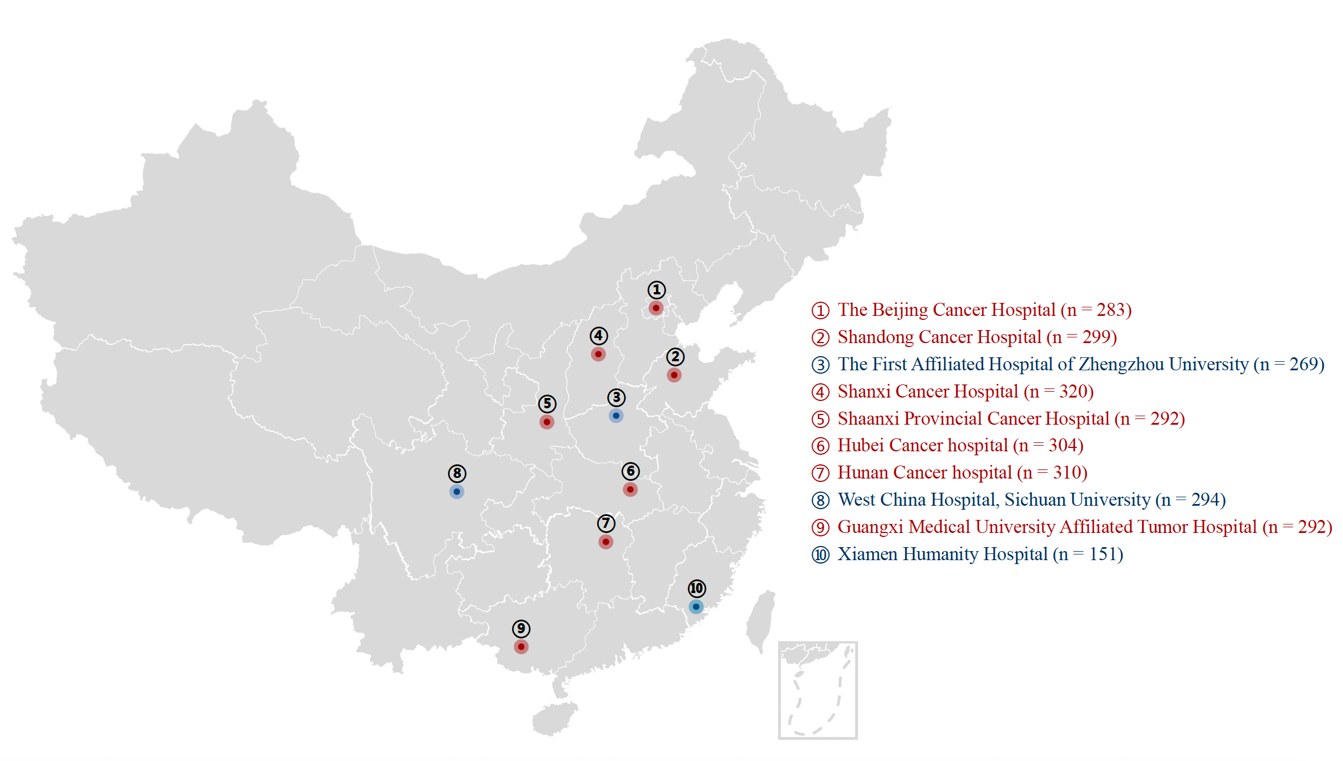


# Figure S2. Flowchart to illustrate the derivation of the analyzed sample.

# Figure S3. Calibration plot for the suicidal ideation risk prediction model in overall patients and patients with different cancer sites.

Patients were evenly classified into ten subgroups based on risk probability. The x-axis represents the predicted mean risk probabilities based on the model. The y-axis indicates the observed mean risk probabilities in the patients. The calibration curve in each cancer site was delineated. A plot lies on the 45-degree line demonstrating the perfect prediction of the model.

#
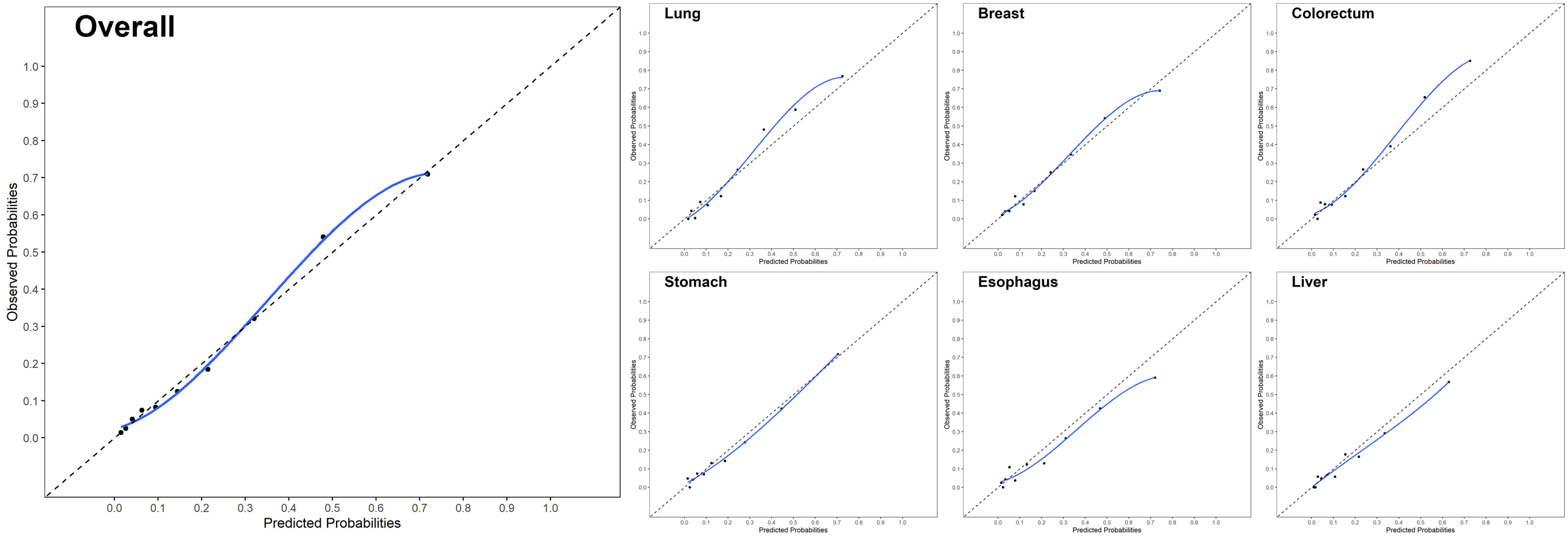


**Figure S4. Online prediction tool with an interactive user-friendly interface for parameter entry and graphic output**


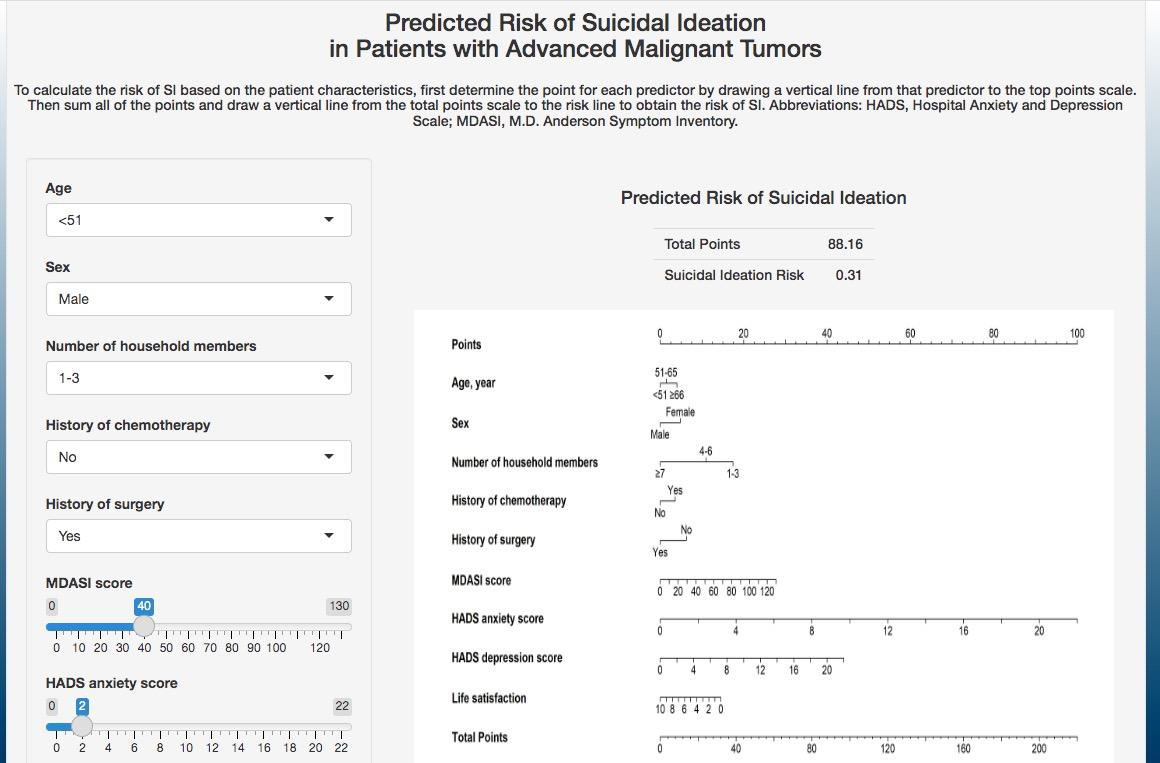

Supplement: Supplementary file 1 — Data S1. [file CAM4-13-e7439-s001.docx]
